# Development of an automated speech recognition interface for personal emergency response systems

**DOI:** 10.1186/1743-0003-6-26

**Published:** 2009-07-08

**Authors:** Melinda Hamill, Vicky Young, Jennifer Boger, Alex Mihailidis

**Affiliations:** 1 The Institute of Biomaterials and Biomedical Engineering, University of Toronto, Toronto, ON, Canada; 2 Intelligent Assistive Technology and Systems Lab, Department of Occupational Science and Occupational Therapy, University of Toronto, Toronto, ON, Canada

## Abstract

**Background:**

Demands on long-term-care facilities are predicted to increase at an unprecedented rate as the baby boomer generation reaches retirement age. Aging-in-place (i.e. aging at home) is the desire of most seniors and is also a good option to reduce the burden on an over-stretched long-term-care system. Personal Emergency Response Systems (PERSs) help enable older adults to age-in-place by providing them with immediate access to emergency assistance. Traditionally they operate with push-button activators that connect the occupant via speaker-phone to a live emergency call-centre operator. If occupants do not wear the push button or cannot access the button, then the system is useless in the event of a fall or emergency. Additionally, a false alarm or failure to check-in at a regular interval will trigger a connection to a live operator, which can be unwanted and intrusive to the occupant. This paper describes the development and testing of an automated, hands-free, dialogue-based PERS prototype.

**Methods:**

The prototype system was built using a ceiling mounted microphone array, an open-source automatic speech recognition engine, and a 'yes' and 'no' response dialog modelled after an existing call-centre protocol. Testing compared a single microphone versus a microphone array with nine adults in both noisy and quiet conditions. Dialogue testing was completed with four adults.

**Results and discussion:**

The microphone array demonstrated improvement over the single microphone. In all cases, dialog testing resulted in the system reaching the correct decision about the kind of assistance the user was requesting. Further testing is required with elderly voices and under different noise conditions to ensure the appropriateness of the technology. Future developments include integration of the system with an emergency detection method as well as communication enhancement using features such as barge-in capability.

**Conclusion:**

The use of an automated dialog-based PERS has the potential to provide users with more autonomy in decisions regarding their own health and more privacy in their own home.

## Background

Falls are one of the leading causes of hospitalization and institutionalization among older adults 75 years of age and older [1,2]. Studies estimate that one in every three older adults over the age of 65 will experience a fall over the course of a year [3,4].

In addition to an overall decline in health, aging is also often accompanied by significant social changes. Many older adults live alone and become isolated from family and friends. Social isolation combined with physical decline can become significant barriers to aging independently in the community, a concept known as aging-in-place [5]. Aging-in-place allows seniors to maintain control over their environments and activities, resulting in feelings of autonomy, well-being, and dignity. In addition to promoting feelings of independence, aging-in-place has also been shown to be more cost-effective than institutional care [6]. However, while aging-in-place is often ideal for both the individual and the public, elders are faced with pressure to move into nursing facilities to mitigate the increased risk of falls and other health emergencies that may occur in the home when they are alone.

Personal emergency response systems (PERSs) have been shown to increase feelings of security, enable more seniors to age-in-place, and reduce overall healthcare costs [7-9]. The predominant form of PERSs in use today consist of a call button, worn by the subscriber on a neck chain or wrist strap, and a two-way intercom connected to a phone line. If help is needed, the subscriber presses the button and a call is placed immediately to a live operator via the intercom. The operator has a dialog with the subscriber, determining the problem and co-ordinating the necessary response, such as calling a neighbour, relative, or emergency response team.

Drawbacks to this approach include the possibility of a high rate of false alarms to the emergency call centre and the subsequent inundation of worried and unsolicited calls to the subscriber. In a study of older women who owned a PERS, many expressed apprehension of unexpected voices and visits from strangers, resenting the need to figure out "why a stranger is talking in my house" and "finding that they show up to check on me" [10]. False alarms typically occur as a result of an accidental button press or failure, on the part of the user, to respond to regularly scheduled check-ins. According to one-call centre manger, false alarms may account for as many as 85% of call-centre calls [11]. False alarms where first responders are sent to the home may further burden limited emergency resources and delay emergency responders from attending to true emergencies. Apart from the worry it may cause family and friends, false alarms may also result in financial losses because of reduced work hours for a friend or relative attending to a false alarm, or resulting from emergency responders having to break down a door or window to get into a home.

Additionally, subscribers to PERSs are not always pleased with the system's usability and aesthetics. Many older adults feel stigmatized by having to wear the push-button activator and current systems place a substantial burden on the subscriber as he/she must remember to wear the button at all times and must be able to press it when an emergency occurs (i.e., the subscriber must be conscious and physically capable) [9]. Finally, some older adults are hesitant to press the button when an emergency does occur because they either downplay the severity of the situation or are wary of being transferred to a long term care facility [8,9].

To circumvent these deficiencies several research groups are exploring the possibility of incorporating PERSs into an intelligent home health monitoring system that can respond to emergency events without requiring the occupant to change his/her lifestyle. Some researchers have devised networks of switches, sensors, and personal monitoring devices to identify emergency situations and supply caregivers and medical professionals with information they need to care for the individual being monitored [12,13]. Through these types of PERSs, the user does not need to wear a physical activator or push anything for an emergency situation to be detected.

One novel technique developed employs computer vision technology (e.g., image capture via video camera) and artificial intelligence (AI) algorithms to track an image of a room and determine if the occupant has fallen [14]. Alternatively, Sixsmith and Johnson [12] used arrays of infrared sensors to produce thermal images of an occupant. The research presented in this paper assumes that a tracking system similar to these will be used to trigger an alarm to the PERS. Regardless of the detection method, once a PERS alarm has been triggered, there is a need to coordinate the response effort with the user. Involving the user allows him/her to maintain control over decisions regarding his/her own health and enables the PERS to provide the appropriate type of response. However, just as with a commercially available push-button triggered PERS, most of the automated PERSs under development immediately connect the user with a call centre when an alarm is triggered [15].

The research described in this paper presents the initial phase of a larger research study investigating the feasibility of using automated dialog and artificial intelligence techniques to improve the usability and efficiency of PERSs for older adults during an emergency situation. In particular, this first phase focuses on demonstrating the possibility of using automatic speech recognition (ASR) with a microphone array and speech recognition software to enable communication and dialog as a means of interfacing with a PERS.

The new generation of ASR technology has achieved significant improvements in accuracy and commercial viability, as demonstrated by their presence in many fields, such as Interactive Voice Response (IVR) telephone systems, medical and business dictation, home and office speech-to-text computer software and others. ASR may be able to provide a simple, intuitive, and unobtrusive method of interacting directly with the PERS, giving the user more control by enabling him/her to chose the appropriate response to the detected alarm, such as dismissing a false alarm, connecting directly with a family member, or connecting with a call centre operator. The following is a description of the prototyping and preliminary testing of an ASR PERS interface, as well as a discussion of other areas within PERS where ASR could provide enhanced information about the state of the subscriber. Although the research described herein does not specifically test with older adult subjects, the results of the research are critical in setting the foundation for future prototype development and testing that will involve older adult subjects.

## Methods

### Development of a dialog-based PERS prototype

**Figure 1:**
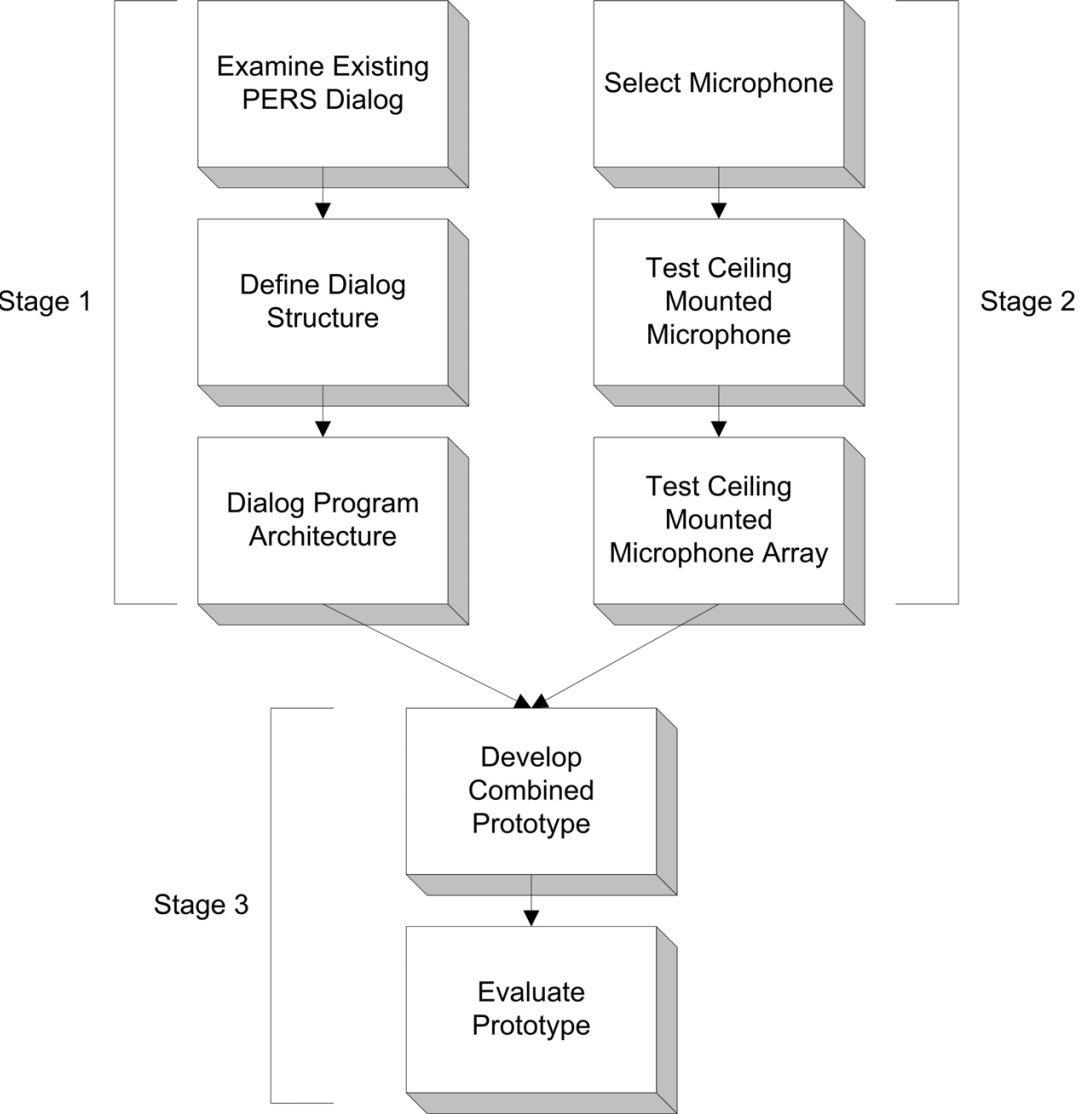
**Prototype development process**. Stage 1 – Definition of dialog and dialog implementation; Stage 2 – Selection and validation of hardware; Stage 3 – Prototyping the PERS interface.

As shown in Figure 1, the development of the prototype occurred with two parallel stages of research. The left branch in Figure 1 (Stage 1) represents the analysis and definition of the dialog that occurs between users and a live call centre in a current, commercially available PERS to develop how the prototype should respond to a detected fall. This includes the selection of software used to run the ASR dialog. The right branch (Stage 2) represents the selection and evaluation of the hardware used for the prototype. The two branches were combined for the building and testing of the prototype (Stage 3).

#### Stage 1 – Definition of dialog and dialog implementation

To promote ease of use and compliance, a goal of this research was to design the automated dialog to be intuitive, effective, and friendly. Since current PERSs have included extensive research on how to interact politely, clearly and efficiently with a subscriber, the dialog for the prototype was based on the existing protocol for the Lifeline Systems Canada call centre. For example, Lifeline operators are instructed to initialise contact with a subscriber with a friendly introduction followed by the open ended question "How may I help you?". The dialog then flows freely until the operator and the subscriber determine together who, if anyone, should be summoned to help.

The need for a dialog is based on the inherent uncertainty about the state of the occupant and about what triggered the alarm. Therefore, the goal of the dialog between the occupant and the PERS is to determine if the alarm is genuine, and if so, the appropriate action to take. To arrive at this goal (i.e., deciding what action to take), the system navigates through a series of verbal interactions resulting in a dialog with the occupant. Different actions available to the prototype are listed in Table 1.

**Table 1:** Actions available to the PERS prototype

**Action Name**	**Action Description**
False Alarm	Accidental alarm – no action needed.

EMS	A call is placed to Emergency Medical Services (EMS).

Responder 1	A contact person from a list that is pre-defined by the user. When compiling this list, the nominated responder is notified and must give consent to respond to emergency calls. Responders can include neighbours, friends, and family.

Responder 2	See description for Responder 1.

Operator	Connect to a live operator. This option can be accessed by the user. It is also the default action the system takes if it does not detect a response from the user or cannot determine which response the user wishes to initialise.

**Figure 2:**
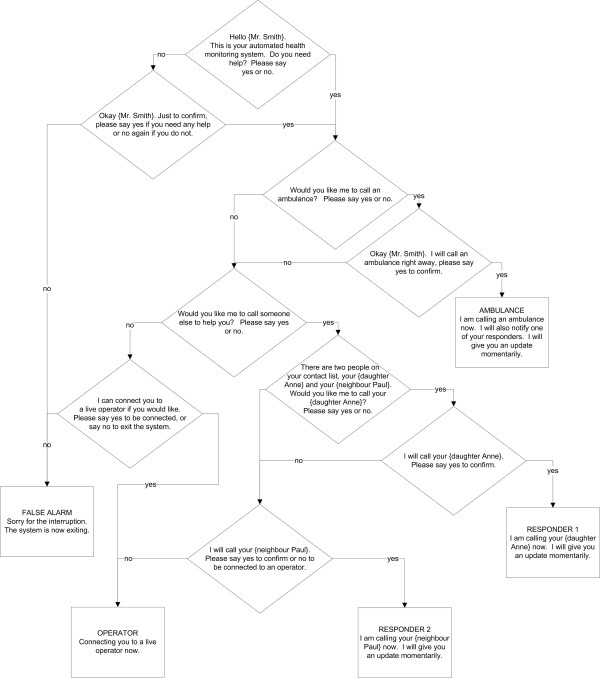
**Flow diagram of system dialog**.

Actions are selected through a dialog exchange between the user and the system. The dialog structure for the prototype is depicted in Figure 2. Human factors experiments conducted on computer voice-based systems have demonstrated highest user satisfaction when automated dialog is modelled after live operators [16]. Thus, the prompts have been developed to emulate the familiar and friendly tone of PERS operators, for example, by the use of personal pronouns ("would you like *me *to call someone else to help you?"), and pre-recording the names of the occupant and responders.

At each dialog node in Figure 2, the corresponding prompt was played over a speaker, then the speech engine was activated to obtain the occupant's answer though a microphone. For these tests, close ended "yes"/"no" questions were selected to create a simple binary tree dialog structure. Transition from one state to the next depended solely on the best match of the user's response to an expression in the grammar (i.e. either 'yes' or 'no'). Each prompt was pre-recorded and saved as separate audio files by the researcher.

When defining the algorithms used to run the user/system dialog, the goal was to create an architecture that would be flexible and adaptable so that it could be easily modified as the project evolved. The modularity offered by modern programming practices and speech application programming interfaces (APIs) allows for flexible and scalable design, and requires minimal rewriting to integrate or remove components at any level. Java Speech API (JSAPI) is a set of abstract classes and interfaces that allow a programmer to interact with the underlying speech engine without having to know the implementation details of the engine itself. Moreover, the JSAPI allows the underlying ASR engine to be easily interchanged with any JSAPI compatible engine [17].

The prototype was tested using the Sphinx 4 speech engine, an ASR written in Java that employs a Hidden Markov Model (HMM) approach for word recognition [18]. The recognition rates for several tests using Sphinx 4 have demonstrated a low word error rate under a variety of testing conditions. Furthermore, this speech engine is open source thus making it easy to use and develop when this application is expanded in the future.

An XML parser was created using Jakarta Commons Digester [19] to load a file containing the dialog and action states (specified in XML format) at runtime. The XML files for the PERS application were built by modifying the Voice XML standard [20], which is generally used for voice enabled web browsing and IVR applications. By implementing the dialogs in separate XML files, the program code does not need to be recompiled in order to change the dialog. This is beneficial for testing different dialogs easily and allows for seamless customization of the system: a dialog for a user in a nursing home (who might want to be prompted for the nursing desk first) could be different from a dialog for a user in the community (who would be asked if they needed an ambulance first). Likewise, the grammar files (in JSGF format) and the prompt files (in .wav file format) were also separated from the code itself to allow for easy modifications. The modular composition of the prototype enables grammars and prompts that take into account the accent or language preference of the user to be deployed on a per-user basis. Indeed, the system can be easily executed with any dialog specified in the XML format.

#### Stage 2 – Selection and validation of hardware

For a speech-based communication system, it is vital that the quality of the user's vocal response is sufficient to be correctly interpreted by the ASR. As such, the choice of microphone is very important. Wearing a wireless microphone is not an ideal solution because, just as with push-buttons, the user must remember and choose to wear the microphone in order to interact with the PERS. Additionally, the user must remember to regularly change the batteries on the wireless device. Ideally an automated PERS should communicate with the user from a distance in a natural fashion, without requiring the user to carry any devices or learn new skills to enable interaction. For this study, the researchers decided that the best location for the microphone would be in the centre of the ceiling of the monitored room as this was out of the way, central to the room, would provide the best sound coverage and could not be easily obstructed.

The close talking microphones typically used for commercial voice recognition applications (e.g., headphones or computer desk microphones) were not appropriate for use in this PERS application since these types of microphones would not be able to capture the occupant's voice with enough strength or clarity. Additionally, single ceiling mounted microphones can suffer from reverberations, echoes in the room, and a variety of background noises (e.g., TVs, radios, dishwashers, etc.) [21,22]. Microphone arrays attempt to overcome such difficulties and have been designed for two purposes: 1) sound localization; and 2) speech enhancement through separation by extracting a target noise from ambient sounds.

The microphone array used in the prototype was custom designed and constructed by researchers at the Department of Computer and Systems Engineering at the University of Carleton in Ottawa, Canada. The array consisted of eight, Electret, unidirectional microphones suspended in an X-shaped configuration. The microphone signal-to-noise ratio was greater than 55 dB, sensitivity was -44 dB (+/- 2 dB) and the frequency response ranged from 100–1000 Hz. A low noise, low-distortion instrumentation amplifier was also built into the array system. The microphone array was mounted on the ceiling in the center of a 16 × 20 ft (4.9 × 6.1 m) room. Four microphones were spaced 10 cm apart along each axis of the array, which was calculated by the researchers from Carleton to be the optimal distance for dimensions of the testing area.

The microphone array described above was designed to specialise in speech enhancement through localisation by implementing delay-and-sum beamforming to enhance audio signals coming from the user and destructively lower the impact of sounds coming from elsewhere [23]. In delay-and-sum beamforming a different delay is calculated for each microphone to account for the time the reference signal needs to travel from a given location to the array. Delay-and-sum beamforming was accomplished by passing the location (presumably known by the PERS) to a Motorola 68 k processor mounted on the array, which used this information to apply the appropriate delay to each microphone. For the prototype, the location of the user was input manually, although it is anticipated that this will be done automatically in a fully functioning PERS as it will be continually tracking the location of the user. This information about the location of the occupant could be used to direct the array to "listen" to the exact spot where the occupant is sitting or laying, making it easier to hear the occupant in both PERS-occupant and human call center operator-occupant dialogs.

#### Test 1 – Performance of a single microphone versus a microphone array with beamforming

The first experiment was designed to test the array in two modes: 1) using a single microphone from the array; and 2) using the array with the beamforming algorithm tuned into a zone of interest.

The AN4 speech database developed by Carnegie Mellon University was selected to test the system. This database has been used in several batch tests throughout the development and evolution of the Sphinx speech engines [24]. The AN4 database has voices from 21 female and 53 male speakers and consists of spoken words and letters. For these tests, only the spoken words were used for a total of 1846 utterances (with 79 unique words).

**Figure 3:**
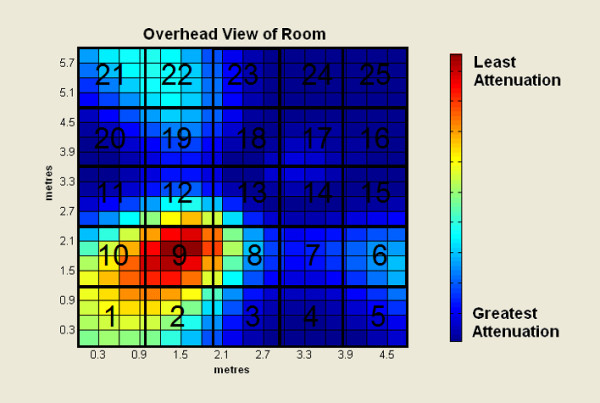
**Attenuation pattern for frequencies of 1850 Hz originating in zone 9**.

Figure 3 illustrates the pattern of attenuation expected from the microphone array for sounds in the mid-range of human speech (1850 Hz) coming from zone 9. AN4 was played over a single computer speaker located on the laboratory floor in zone 9 for each test. Neither the speaker's location nor volume changed during the tests. For the single microphone test, only one microphone on the array was turned on. In the case of the beamforming tests, all the microphones were used and the researcher manually entered the location of the AN4 speaker. To create ambient noise interference, a pre-recorded audio track of a bubbling kettle (with a signal to noise ratio (SNR) of approximately 6.7 dB) was played over a separate speaker. The kettle noise was played from zone 17, the spot that caused the most destructive interference with the AN4 speaker. The Sphinx 4 ASR was used to analyse both sets of tests. The output from the ASR was compared with the known data to determine recognition rates.

#### Test 2 – Testing "yes"/"no" word recognition rate

While the results from the beam-forming tests were conducted with a large vocabulary, it is hypothesised that ASR recognition would improve significantly with a simple two-word vocabulary consisting of "yes" and "no". A convenience sample of nine subjects, 4 male and 5 female, was used for this experiment. The subjects ranged from 20 to 30 years of age. Each subject was asked to sit in the same spot as the AN4 speaker used in the previous tests (depicted as zone 9 in Figure 3). The subject was asked to speak at their normal volume and say the words 'yes' and 'no' twice for three conditions for a total of twelve utterances per subject (108 words in total). The three conditions were: 1) bubbling kettle interference played in the same location as previous tests (zone 17 in Figure 3 – area of the most attenuation of the human voice); 2) bubbling kettle interference played directly under the array (zone 13 in Figure 3 – intermediate attenuation); and 3) no noise interference.

#### Stage 3 – Prototyping the PERS interface

**Figure 4:**
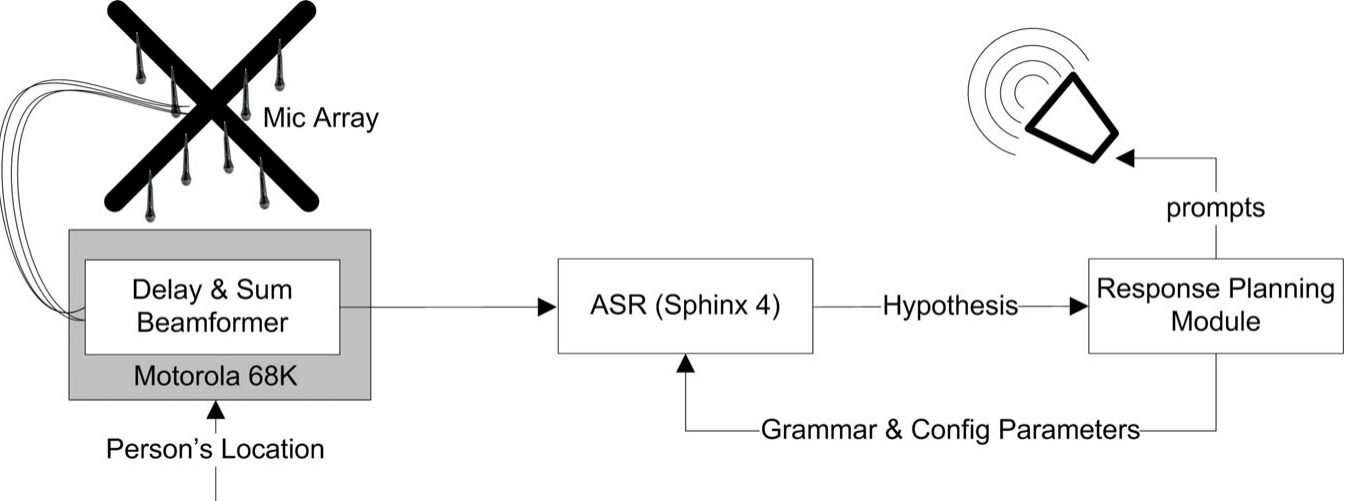
**Prototype Architecture**.

The dialog system developed in Stage 1 and microphone array selected and tested in Stage 2 were combined into the architecture depicted in Figure 4. The response planning module executes the dialog and actions outlined in Figure 2. Pre-recorded actions selected by the system were played over the speaker. In this system only audio files were played, however in a working system a call would also be placed to the appropriate party.

#### Test 3- Efficacy of the prototype dialog

This test examined the overall efficacy of the prototype automated PERS dialog interface. A convenience sample of four subjects (3 male and 1 female, healthy and between the ages of 20 and 30) each conducted a set of three scenarios with the system, for a total of 12 dialogs. Before each dialog, the subject was asked to envision a scenario read to them by the researcher and then asked to interact with the prototype to get the recommended assistance. The three scenarios were: 1) they were injured and needed an ambulance, 2) they had fallen, but only wanted their daughter to come and 3) a false alarm.

The Response Planning Module employed the dialog structure outlined in Figure 2, and the ASR matched the subjects' responses to either yes or no.

## Results

Table 2 presents the recognition results for a single microphone versus beamforming using the AN4 database (Test 1).

**Table 2:** Results of AN4 batch tests at SNR of ~6.7 dB

	**Single Microphone**	**Beamforming**
Words Played	1846	1846

Total Errors	1302	924

*Substitutions*	*401*	*371*

*Insertions*	*16*	*7*

*Deletions*	*885*	*546*

Accuracy	29.5%	49.9%

As seen in Table 2, tests showed about a 20% improvement in accuracy when beamforming was used, demonstrating that a microphone array using basic delay-and-sum beamforming provides improved recognition results over a single microphone in the presence of moderate volume interference noise. After obtaining these results, further tests were performed at a SNR of approximately 0dB and resulted in no recognition by either the single microphone or the array.

The results of the yes/no recognition test (Test 2) are summarised in Table 3. There were no errors in the no noise condition, six errors when the noise was directly under the microphone and four errors in the zone of previous tests. As the accuracy of this test was significantly higher than the AN4 test, it was decided that the prototype dialog questions would follow a closed-ended, "yes"/"no" format.

**Table 3:** ASR Yes/No vocabulary recognition results

	**Scenario 1**	**Scenario 2**	**Scenario 3**	**Overall**
Words read	36	36	36	108

Total Errors	4	6	0	10

Accuracy	89%	83%	100%	93%

When the prototype dialogue was tested through the use of scenarios (Test 3), all 12 tests concluded with the system selecting the desired action, despite a word error rate of 21% (11 errors in 52 words spoken). The reason for this was because the system confirmed the user's selection before taking an action (see Figure 2). The errors consisted of three substitutions (yes for no or visa-versa) and eight deletions (missed words). Most of the deletions were missed by the ASR because users were speaking their response while the message was still being played by the system.

## Discussion

The results from tests with the prototype are encouraging. During the array testing, simple delay-and-sum beamforming resulted in a considerable improvement (20%) in the word recognition rate of the array over a single microphone. This improvement might be greater with more complex microphone array algorithms [25,26] and pre-filters [22]. Additionally, further experimentation with the Sphinx 4 configuration parameters may result in increased ASR performance [27].

The "yes"/"no" tests have twofold results. Firstly, unsurprisingly the location of noise interference has an impact on the ASR's ability to correctly identify words. This suggests that the system performance will be affected by the location and presence of unwanted noise. Secondly, the reduction of the users' response to either "yes" or "no" greatly improves ASR recognition. In this case, overall recognition rates for the beam-former increased from about 50% to 90%. This increase is very likely the result of the significant simplification of possible matches the ASR had to choose from. However, it must be taken into consideration that the AN4 tests were conducted by playing the database over (high quality) speakers, while the 'yes'/'no' tests involved live humans.

The full prototype test conducted in Stage 3 (Test 3), resulted in several important insights. First, although all of the errors made in Test 3 were corrected by the confirmation-nature of the dialog, there is still the possibility (4.5% given a word error rate of 21%) that 2 errors could occur in sequence, resulting in the PERS making the wrong decision. This is an unacceptably high error rate as the occupant must always be able to get help when it is needed. As such, there needs to be a method (or methods) that the occupant can use to activate or re-activate the system whenever s/he wishes. One option is to enable a unique "activation phrase" that the user selects during system set-up. When the user utters this activation phrase, a dialog is initiated, regardless of whether or not an emergency has been detected. To further improve system accuracy, information from a vision system tracking the occupant could be used to reduce uncertainty about a situation. For example, if the user is lying still on the floor, this information could increase the weighting across possible answers that lead to emergency actions as opposed to false alarms. This type of intelligent, multi-sensor fusion can be achieved though a variety of planning and decision making methods such as partially observable Markov decision processes (POMDPs) [28]. Regardless, it is vital that in the case of doubt about a user's response (or lack thereof), the system should connect the user to a live operator, thus ensuring that the user's safety is maximised.

Secondly, the test subjects in Test 3 quickly became accustomed to how the system worked and would often start responding while the system was still "speaking". As the microphone was not activated until after the system finished playing a prompt (so as to avoid the system interpreting its' own prompt as a user response), these responses were missed and would have to be repeated, causing some confusion and frustration. This highlights the necessity for the user to be able to "barge-in" while a prompt is in progress. This is especially important in a system designed for emergency situations, where the user may be familiar enough with the system to anticipate the last few words in a system dialog and may be too panicked or in pain to wait. Most telephone voice systems today have taken this property of dialog into account, and allow users to speak before the system has completed its side of the dialog (i.e. barge-in), however the separation of the phone earpiece and receiver makes this approach easier to implement over the telephone than it would be for the type of PERS described here. Nevertheless, it is an important feature that will be investigated in future designs.

The literature has conflicting opinions on the comfort of seniors with recorded voices [10,29]. There is also a lack of evidence on whether an automated system would be appropriate for emergency situations where users may be under duress. Further research is needed to determine whether a recorded voice would quell or create confusion and/or discomfort and also whether occupants can attend to a series of directed questions while in a crisis. Additionally, tests with older adults would provide feedback in terms of usability and acceptability. As older adults represent the majority of targeted users of this technology, these questions must be well investigated and answered with the intended user population.

Finally, it must be stressed that although this paper presents promising preliminary research towards a new alterative to the current PERS techniques, more research is necessary to improve interactions with the user and to make the system more robust. While false positives (i.e., false alarms) can be annoying and costly, false negatives (i.e., missed events) must never occur as this could place the life of the occupant in jeopardy. Testing involving different software, hardware, and environment choices, using larger, more comprehensive groups of test subjects is needed. Only after such extensive testing with subjects in real-world settings will dialog interface technology be ready for the mass market.

Although the dialog program architecture for this prototype is fairly simple and deterministic, it was created with a modular architecture into which other algorithms could be easily applied. For instance, by using appropriate abstract classes and implementations, methods such as decision theoretic planning, such as a Markov decision process (MDP) [30] or POMDP [11] based approach, could be applied in the future to converge on dialogs that were most effective for each particular user.

In general, this prototype demonstrates the improved ability of a microphone array to remove noise from the environment compared to a single microphone. This enhances ASR accuracy and also allows for easier communication between a call centre representative and the occupant. Importantly, the successful recognition of most false alarms could significantly reduce false alarm call volumes in current PERS call centres, allowing operators to focus on real emergencies.

## Limitations

Hearing loss is extremely common among seniors [31] and the loud volume settings on TVs and radios could lead to zero or even negative SNR. Therefore, before it can be implemented in a home environment, improvements in ASR performance will be needed to ensure the PERS interface is robust with smaller SNR, as well as non-uniform noise that contains human speech (e.g. TV, radio).

These tests were limited in the type of voice samples used. The system was tested with users under calm, casual circumstances. It will be important to conduct tests on voices in emergency situations, either live or using recorded conversations from call centres, in order to ensure speech recognition performance is upheld when a person may be shaken by a fall or other crisis in the home. Secondly, these experiments were limited to a younger adult sample. It is important that tests be run with older adults on a system that has been trained using a database of older adult voices. The authors are currently working to build such a database. Limited work in comparing the success rate of ASR for various age groups indicates that differences may exist [32,33]. Finally, tests should also be conducted with people of different backgrounds who have strong accents to assess the affect on accuracy and determine the extent of customizations that would be needed [34].

## Conclusion

Implementing ASR in the domain of PERS is a complex process of investigating and testing many tools and algorithms. The modularity of the code and of the components used in this study will facilitate the optimisation of the ASR and microphone array parameters, the addition of more complex dialog states, and the potential addition of statistical modelling methodologies, such as techniques involving planning and decision making.

Although the prototype did not perform perfectly, accuracy was significantly improved by limiting the vocabulary to 'yes' and 'no'. By including a confirmation for each action that the system was about to take, the prototype was able to overcome errors and successfully determine the proper action for all test cases. As such, the prototype designed and tested in this study demonstrates promising potential as a solution to several problems with existing systems. Notably, it provides a simple and intuitive method for the user to interact with PERS technology and get the type of assistance he/she needs. Having an automated, dialog-based system provides the occupant with more privacy and more control over decisions regarding one's own health. Additionally, the microphone array system proposed in this research requires only one device to be installed per room in the home or apartment. If coupled with automatic event detection, such as a computer vision-based system, this would be much simpler to install and maintain than other proposed automated PERSs, which generally use a multitude of sensors or RFID tags throughout the home. These advantages would likely translate into a significant reduction in non-compliance, as greater burden would be transferred from the user to the technology.

The next phase of research is currently underway and is focused on improving the robustness of the automated dialog-based and intelligent PERS specifically for older adults. An older adult speech corpus containing emergency type speech in Canadian English is being developed for this purpose. Once completed, this older adult speech corpus will be used to train the ASR component of the prototype PERS. We hypothesize that an ASR system trained with older adult speech in-context will be more effective than an ASR system trained with non-older adult speech out-of-context. In addition, older adult voices will be recorded in mock emergency situations and will be used to test the prototype PERS system. The decision making and dialogue capability of the automated PERS will also be further refined and tested possibly with a slightly larger vocabulary (e.g., help, ambulance), a probabilistic decision-making model, and/or a more complex language model. To enhance system flexibility, the ability to barge-in at any time is also being explored. Once the system is operational, quantitative and qualitative system and usability testing with older adult subjects will be conducted.

## Competing interests

The authors declare that they have no competing interests.

## Authors' contributions

MH carried out the experiments, background research, analysis and interpretation of the results and drafting of the article. AM conceived of the study and participated in the concept development, testing and literature survey. VY participated in the background research, drafting of the article and is performing the next phase of the research. JB assisted with the system design and testing, data analysis, and drafting the article. All authors have read and approved the final manuscript.
